# Access to medicines for chronic diseases in Brazil: a multidimensional approach

**DOI:** 10.1590/S1518-8787.2016050006161

**Published:** 2016-12-01

**Authors:** Maria Auxiliadora Oliveira, Vera Lucia Luiza, Noemia Urruth Leão Tavares, Sotero Serrate Mengue, Paulo Sergio Dourado Arrais, Mareni Rocha Farias, Tatiane da Silva Dal Pizzol, Luiz Roberto Ramos, Andréa Dâmaso Bertoldi

**Affiliations:** I Departamento de Política de Medicamentos e Assistência Farmacêutica. Escola Nacional de Saúde Pública Sérgio Arouca. Fundação Oswaldo Cruz. Rio de Janeiro, RJ, Brasil; II Departamento de Farmácia. Faculdade de Ciências da Saúde. Universidade de Brasília. Brasília, DF, Brasil; III Programa de Pós-Graduação em Epidemiologia. Faculdade de Medicina. Universidade Federal do Rio Grande do Sul. Porto Alegre, RS, Brasil; IV Departamento de Farmácia. Faculdade de Farmácia, Odontologia e Enfermagem. Universidade Federal do Ceará. Fortaleza, CE, Brasil; V Departamento de Ciências Farmacêuticas. Centro de Ciências da Saúde. Universidade Federal de Santa Catarina. Florianópolis, SC, Brasil; VI Departamento de Produção e Controle de Medicamentos. Faculdade de Farmácia. Universidade Federal do Rio Grande do Sul. Porto Alegre, RS, Brasil; VII Departamento de Medicina Preventiva. Escola Paulista de Medicina. Universidade Federal de São Paulo. São Paulo, SP, Brasil; VIII Departamento de Medicina Social. Faculdade de Medicina. Universidade Federal de Pelotas. Pelotas, RS, Brasil

**Keywords:** Adult, Aged, Drug Utilization, Chronic Disease, Socioeconomic Factors, Health Surveys

## Abstract

**OBJECTIVE:**

To analyze the access to medicines to treat non-communicable diseases in Brazil according to socioeconomic, demographic, and health-related factors, from a multidimensional perspective.

**METHODS:**

Analysis of data from the National Survey on Access, Use and Promotion of Rational Use of Medicines (PNAUM), household survey, sampling plan by conglomerates with representativeness of the Brazilian population and large areas of the country, according to sex and age domains. Data collected in 2013–2014 with sample of adults (≥ 20 years) who reported having non-communicable diseases and medical indication for use of medicines (n = 12,725). We assessed the prevalence of access to medicines for self-reported non-communicable diseases, considering four dimensions: availability, geographic accessibility, acceptability, and affordability. We applied Pearson’s Chi-square test to assess the statistical significance of the differences between strata, considering the level of significance of 5%. We found prevalence of 94.3%, 5.2%, and 0.5% for full, partial, and null access, respectively. Higher prevalence was observed among seniors in the South compared to the Northeast; for those who reported having one non-communicable disease compared to those who reported having two or more; for those who needed one medicine compared to those who needed three or more; and for those who self-assessed their health as good or very good. Geographic accessibility was similar in the Unified Health System and in the private pharmacies (72.0%). Total availability of medicines was 45.2% in the Unified Health System, 67.4% in the Popular Pharmacy Program, and 88.5% in private pharmacies. Acceptability was 92.5% in the Unified Health System, 97.8% in the Popular Pharmacy Program, and 98.7% in private pharmacies. As to affordability, 2.6% of the individuals failed to take the medicines they should in the 30-day period prior to the interview due to financial difficulty. Prevalence of full access to medicines for non-communicable diseases in Brazil is high and presents significant differences for age group, region of the country, number of non-communicable diseases, and for medicines prescribed and self-assessment of health. The major barriers to access to medicines were identified in the dimensions analyzed.

## RESUMO

**OBJETIVO:**

Analisar o acesso a medicamentos para tratar doenças crônicas não transmissíveis no Brasil segundo fatores socioeconômicos, demográficos e de saúde, sob perspectiva multidimensional.

**MÉTODOS:**

Análise de dados da Pesquisa Nacional sobre Acesso, Utilização e Promoção do Uso Racional de Medicamentos (PNAUM), inquérito domiciliar, plano amostral por conglomerados com representatividade da população brasileira e grandes regiões do País, segundo domínios de sexo e idade. Dados coletados em 2013-2014 com amostra constituída por adultos (≥ 20 anos) que referiram ter doenças crônicas não transmissíveis e indicação médica para usar medicamentos (n = 12.725). Avaliou-se a prevalência de acesso aos medicamentos para doenças crônicas não transmissíveis autorreferidas, considerando quatro dimensões: disponibilidade, acessibilidade geográfica, aceitabilidade e capacidade aquisitiva. Aplicou-se teste Qui-quadrado de Pearson para avaliar a significância estatística das diferenças entre os estratos, considerando o nível de significância de 5%.

**RESULTADOS:**

Foram encontradas prevalências de 94,3%, 5,2% e 0,5% para acesso total, parcial e nulo, respectivamente. Maiores prevalências ocorreram entre os idosos, na região Sul comparada à região Nordeste; naqueles que referiram ter uma doença crônica não transmissível comparados aos que referiram ter duas ou mais; naqueles que precisavam de um medicamento comparados aos que precisavam de três ou mais; e naqueles que autoavaliaram sua saúde como boa ou muito boa. A acessibilidade geográfica foi semelhante no Sistema Único de Saúde e nas farmácias privadas (72,0%). A disponibilidade total de medicamentos foi de 45,2% no Sistema Único de Saúde, 67,4% no Programa Farmácia Popular e 88,5% nas farmácias privadas. A aceitabilidade foi de 92,5% no Sistema Único de Saúde, 97,8% no Programa Farmácia Popular e 98,7% nas farmácias privadas. Quanto à capacidade aquisitiva, 2,6% dos indivíduos não tomou os medicamentos que deveria nos 30 dias anteriores à entrevista devido à dificuldade financeira.

**CONCLUSÕES:**

A prevalência do acesso total aos medicamentos para doenças crônicas não transmissíveis no Brasil é alta e apresenta diferenças significativas por faixa etária, região do País, número de doenças crônicas não transmissíveis e de medicamentos prescritos e autoavaliação da saúde. Foram identificadas as principais barreiras ao acesso a medicamentos nas dimensões analisadas.

## INTRODUCTION

Medicines save lives and improve the health of people, preventing, curing, or controlling and reducing morbidity and mortality associated with acute and chronic diseases^2^. Access to medicines is a fundamental human right^11^ and results from the interaction of a complex network of processes, events, actors and their interests, including research and development institutions, chemical and pharmaceutical industries, regulatory agencies, health systems and health services, which also includes the medicine user^10^ ^,^ ^15^.

In addition to access to health services for medical consultation, diagnosis and prescription, the effective access to medicines depends on their physical availability in pharmacy; on the users’ geographic accessibility to pharmacy services; on the users’ acceptability in relation to pharmacy services; and on the affordability of providers or individuals and families^5^ ^,^ ^14^. Study that discusses the main theoretical models prescribed in the literature to analyze the access to medicines in the level of health services recognizes these dimensions as core^4^.

In Brazil, the non-communicable diseases (NCD) are the main source of burden of disease, disproportionately affect the poorest populations, and were responsible for 72.0% of the mortality in 2011^16^. The rapid demographic transition, with an increase in the relative weight of adults and seniors in the population pyramid, indicates trend of increasing for the burden of these diseases and for consumption of medicines in the country^23^. Control of these diseases and of their risk factors depends on a set of health actions, which includes timely health care and adequate provision of medicines^16^. In Brazil, besides the private market supply, these medicines are available for free in public health services (SUS), including primary health care^7^ and pharmacies of the Popular Pharmacy Program (FPFP)^24^. This program is a strategy to scaling up access, in which a set of medicines is supplied for free and another, broader, is sold at subsidized prices^a^.

Household surveys of national scope that included questions about access to medicines, such as the World Health Survey^8^ (2003) and the Assessment of Pharmaceutical Services in Brazil^19^ (2005), reported prevalence of access to medicines from 87.0% to 89.0%. The National Health Survey (2013) reports lower prevalence for global access (82.5%)^12^. Study on access to medicines of continuous use with women with NCD in Brazil found an overall prevalence of access of 87.0%^13^.

Study with adults and seniors with NCD in two regions of the country^20^ reported general prevalence of access to medicines of continuous use of 78.8% in the Northeast and 83.7% in the South. Study with senior population in Brazil who reported long-term use of medicines showed prevalence of full access of 86.0%^27^.

No studies with national representativeness were found that detailed the access to medicines for the set of most prevalent NCD in Brazil, according to the four dimensions proosed in this study.

Considering the international commitment to reduce morbidity and mortality by NCD and the efforts developed to meet these goals^16^, it is necessary to analyze if the increasing government spending with medicines resulted in effective access to them by the Brazilian population. The possibility of addressing the issue according to the dimensions of access enables identifying weak points in the current pharmaceutical services policy in the country and proposing measures to better guide improvement processes.

This study aimed to analyze the access to medicines to treat non-communicable diseases in Brazil according to socioeconomic, demographic, and health-related factors, from a multidimensional perspective.

## METHODS

The household survey component of the National Survey of Access, Use, and Promotion of Rational Use of Medicines (PNAUM) was a population-based study with cross-section design and sampling plan by conglomerates with representativeness of the Brazilian population and of large regions of the country, according to sex and age domains. Data collection was conducted from September 2013 to February 2014 with face-to-face interviews in households and application of questionnaires. The instruments used were developed by a group of Brazilian researchers specialized on the area^b^ and the data collected was stored in electronic device (tablet PC). Further details about the sampling and about the data collection logistics are described in the methodological article of the PNAUM^18^.

This study considered a sample of adults aged 20 years or older who reported having some NCD diagnosed by physician: “Has a physician ever told you that you have hypertension or high blood pressure and medical indication for use of medicines?”. This question was repeated for each chronic disease investigated in the study (n = 12,725). Access to medicines is the outcome variable of this study.

A group of NCD (hypertension or high blood pressure; cardiovascular diseases, chronic obstructive pulmonary disease, diabetes, arthritis, depression) was investigated by applying a specific questionnaire, which included questions about the prior existence of other diseases for six months or more at the time of the interview. Data collection also considered aspects related to access in the main sources of supply of medicines of Brazil, which are: pharmacies of the Unified Health System (FSUS) public network; pharmacies of the Popular Pharmacy Program: public network and private pharmacies (FPFP), and private pharmacies in general (Fprivadas).

The independent variables analyzed were: sex (female; male); age group (20-39; 40-59; 60 years old or older); educational level in completed school cycles (0-4; 5-8; 9-11; 12 years or more); economic classification (A/B; C; D; E) according to the Brazil Economic Classification Criterion, developed by the Brazilian Association of Survey Companies (CCEB 2013/ABEP – http://www.abep.org/); geographical region of residence (North; Northeast; Southeast; South, Midwest); number of NCD reported (1; 2; 3 or more); number of medicines needed (1; 2; 3-4; 5 or more) and self-assessment of health (very good or good; regular; bad or very bad). The age group cut-off points were determined by the sampling domains.

The theoretical model employed was prescribed by Penchansky and Thomas^22^ and adapted by Luiza and Bermudez^14^ to evaluate the access to medicines. This model assumes that access to medicines results from the interaction of four dimensions: (i) physical availability: relation between type and quantity of medicines needed and type and quantity of products supplied; (ii) affordability: relation between prices of medicines and capacity to pay for them; (iii) geographical accessibility: relation between the location of medicines providers and the location of the user; (iv) acceptability: fit between characteristics of products and services and expectations and needs of users (Figure).

**Figure:**
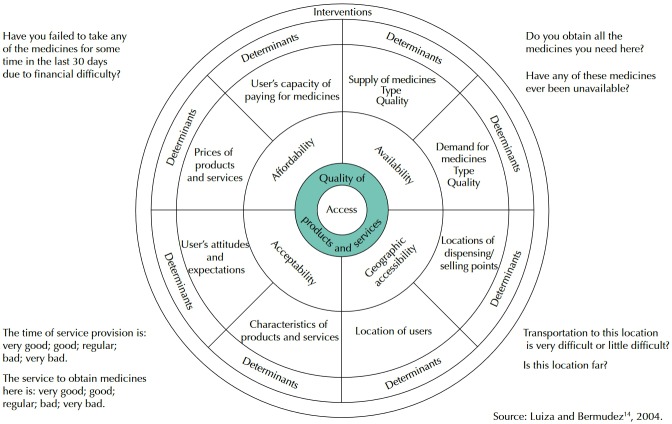
Logical model of access to medicines and questions that guided the construction of the indicators to evaluate each of the dimensions. PNAUM, Brazil, 2014.

Access to medicines for NCD was studied with all individuals who reported having medical indication for medicine (n = 12,725) based on the question: “In the last 30 days, have you been out of any of these medicines for some time?”. Those who reported having obtained all medicines prescribed by the physician were considered with full access to treatment. Partial access, whenever the user declared to have obtained some of the medicines prescribed by the physician or to have been out of some of those that should have been obtained in the last 30 days due to unavailability on FSUS or to financial difficulty; and null access, whenever the user declared to have obtained none of the medicines prescribed by the physician in the last 30 days due to unavailability on FSUS or to financial difficulty.

Indicators of the first three dimensions of access described below, all dichotomously categorized, were calculated for each source of provision of medicines investigated (FSUS, FPFP, and Fprivadas) when the respondent declared to be user of one of them, that is, had obtained at least one of the medicines prescribed by the physician at the source. Geographic accessibility was estimated considering the proportion of users who obtained at least one medicine prescribed by the physician and reported that the location was neither difficult to reach nor far. Total availability was considered whenever the user declared to have obtained all the medicines prescribed in the source considered and that no medicine had ever been unavailable. Acceptability was considered whenever the user assessed the hours of operation and the quality of the service they used. The question offered the possibilities of the Likert scale with five points, re-categorized into: very good or good; regular; bad or very bad. Affordability was considered whenever the respondent declared having been out of one of the medicines prescribed by the physician in the last 30 days due to financial difficulty. Analysis of the four dimensions aims to identify potential barriers that users of the pharmacy services are facing to obtain access to medicines for NCD prescribed by the physician.

The data were stored and analyzed in the program SPSS, v. 20.0 for Windows (SPSS Inc., Chicago, IL, USA), using the CSPLAN command set suitable for the analysis of complex samples and ensuring the necessary sample weighting. For all variables we estimated percentages and their 95% confidence intervals and applied Pearson’s Chi-square tests to assess the statistical significance of the differences between strata, considering the level of significance of 5%.

The project was approved by the National Commission of Ethics in Research (CONEP – Protocol 18947013.6.0000.0008) and by the Research Ethics Committee of the Universidade Federal do Rio Grande do Sul (Protocol 19.997). All interviews were conducted only after the informed consent form was read and signed by the respondent or legal guardian in the case of individuals unable to answer their own questionnaire and those aged under 15 years.

## RESULTS

Most persons (94.3%) reported having full access to medicines for the NCD under analysis, with some significant differences in the categories measured. Full access was higher: among those aged over 60 years; in the South, compared to the Northeast; among those who reported having one NCD; and among those who needed one medicine and self-assessed their health as very good or good (Table 1).

**Table 1:** Access to medicines for non-communicable diseases in adults and seniors (≥ 20 years), according to socioeconomic, demographic, and health-related variables. PNAUM, Brazil, 2014.

Variable	Prevalence of access to medicines for NCD						

	Full		Partial		Null		p^b^
		
	%^a^	95%CI	%^a^	95%CI	%^a^	95%CI	
Sex							0.025
Male	95.6	94.3–96.6	3.9	2.9–5.2	0.5	0.3–0.9	-
Female	93.6	92.4–94.6	5.8	4.9–7.0	0.6	0.4–0.8	-
Age group (years)							< 0.001
20-39	91.2	87.5–93.8	7.5	4.9–11.3	1.3	0.8–2.3	-
40-59	93.5	92.2–94.6	5.8	4.7–7.0	0.7	0.5–1.1	-
≥ 60	96.2	95.3 –96.9	3.7	3.0–4.6	0.1	0.04–0.1	-
Educational level^c^							0.032
0-4	95.1	93.9–96.0	4.4	3.5–5.5	0.5	0.3–0.9	-
5-8	95.1	93.7–96.3	4.5	3.4–5.9	0.4	0.2–0.7	-
9-11	92.6	90.7–94.2	6.8	5.2–8.7	0.6	0.3–1.1	-
≥ 12	95.1	92.7–96.7	4.3	2.7–6.6	0.7	0.3–1.6	-
Region							< 0.001
North	93.6	91.1–95.4	4.7	3.2–6.9	1.7	0.9–3.2	-
Northeast	92.0	90.2–93.5	6.8	5.4–8.6	1.2	0.8–1.9	-
Southeast	94.9	93.4–96.1	4.8	3.7–6.4	0.3	0.1–0.6	-
South	95.8	94.4–96.9	3.9	2.9–5.2	0.3	0.1–0.8	-
Midwest	93.9	92.2–95.2	5.8	4.5–7.3	0.3	0.1–0.8	-
CCEB^d^							0.004
A/B	96.4	94.7–97.5	3.2	2.1–4.9	0.4	0.1–1.0	-
C	94.1	93.0–95.1	5.5	4.5–6.6	0.5	0.3–0.7	-
D	92.8	90.5–94.6	6.1	4.5–8.3	1.1	0.6–2.0	-
E	90.8	85.3–94.3	8.7	5.2–14.2	0.5	0.2–1.4	-
Number of NCD							< 0.001
1	96.7	95.7–97.5	2,5	1.8–3.5	0,7	0.5–1.1	-
2	93.5	91.7–94.9	5,9	4.6–7.6	0,6	0.3–1.1	-
≥ 3	91.3	89.7–92.8	8,5	7.1–10.1	0,2	0.1–0.4	-
Number of medicines needed^e^							< 0.001
1	97.0	95.7–98.0	2.7	1.8–4.0	0.3	0.1–0.6	-
2	94.4	92.7–95.7	5.5	4.2–7.2	0.2	0.04–0.7	-
3-4	94.2	92.4–95.5	5.8	4.5–7.6	-		
≥ 5	91.1	88.4–93.2	8.9	6.8–11.6	-		
Self-assessment of health							< 0.001
Very good/Good	96.5	95.5–97.3	3.1	2.3–4.1	0.4	0.2–0.7	-
Regular	93.2	91.8–94.3	6.2	5.1–7.6	0.6	0.4–1.0	-
Bad/Very bad	86.8	83.6–89.4	12.4	9.8–15.5	0.8	0.4–1.8	-
All	94.3	93.4–95.1	5.2	4.4–6.0	0.5	0.4–0.7	-

NCD: non-communicable diseases

^a^ Percentage adjusted by sample weights and by post-stratification according to age and sex.

^b^ Pearson’s Chi-squared test.

^c^ In completed grades in school.

^d^ According to the 2013 Brazil Economic Classification Criterion (CCEB 2013) of the Brazilian Association of Research Companies (ABEP). Available from: http://www.abep.org

^e^ Medicines prescribed by the physician.

Geographic accessibility was similar for FSUS (72.3%) and FPrivadas (72.4%) and lower for FPFP (67.3%), but with no significant differences (Table 2). Individuals who reported better accessibility to the FSUS were those residing in the South compared to the North and Midwest, those who needed one medicine compared to those who needed five or more, and those who assessed their health as very good or good compared to those who reported worse self-perception of health. For FPFP, geographic accessibility was better in the Southeast compared to the North, for individuals belonging to class E compared to class D, for those who reported one NCD compared to those who reported three or more NCD, for those who needed one or two medicines compared to those who needed five or more, as well as for individuals with better self-perception of health. For Fprivadas, better geographic accessibility was reported by those belonging to class A/B compared to class D, by those who needed two medicines compared to those who needed five or more medicines, and by those who self-assessed their health as very good or good compared to bad or very bad (Table 2).

**Table 2:** Geographic accessibility to the SUS pharmacy services, Popular Pharmacy Program and private pharmacy, according to socioeconomic, demographic, and health-related variables. PNAUM, Brazil, 2014.

Variable	Geographic accessibility to pharmacy services					

	SUS pharmacy		Popular pharmacy		Private pharmacy	
		
	%^a^	95%CI	%^a^	95%CI	% ^a^	95%CI
Sex	0.007^b^		0.064^b^		0.165^b^	
Male	75.3	71.4–78.8	70.8	65.6–75.6	73.9	70.3–77.1
Female	70.7	67.9–73.4	65.5	61.3–69.4	71.7	68.9–74.3
Age group (years)	0.057^b^		0.151^b^		0.110^b^	
20-39	67.4	61.7–72.6	60.0	50.0–69.3	68.3	62.5–73.7
40-59	74.1	70.5–77.3	68.5	63.5–73.0	73.3	69.9–76.4
≥ 60	72.0	68.6–75.3	68.1	64.4–71.6	73.2	70.5–75.8
Educational level^c^	0.597^b^		0.643^b^		0.898^b^	
0-4	71.8	67.6–75.6	69.3	64.4–73.8	73.2	67.9–76.2
5-8	73.2	69.6–76.4	65.6	59.8–71.0	72.6	68.3–76.6
9-11	71.2	67.7–74.6	66.3	60.9–71.3	71.8	68.5–74.8
≥ 12	74.7	69.3–79.4	66.8	58.9–73.7	72.2	66.7–77.1
Region	0.012^b^		0.005^b^		0.140^b^	
North	59.5	53.5–65.3	54.3	47.0–61.4	69.4	64.2–74.1
Northeast	74.4	69.8–78.5	61.1	54.9–66.9	69.6	64.5–74.3
Southeast	71.6	66.8–75.9	70.8	64.8–76.1	75.2	70.5–79.3
South	77.1	73.0–80.8	69.4	63.4–74.8	71.3	66.2–75.9
Midwest	67.0	61.1–72.4	59.1	51.5–66.4	68.3	62.4–73.7
CCEB^d^	0.162^b^		0.001^b^		0.001^b^	
A/B	75.4	71.1–79.2	73.7	67.6–79.0	77.8	74.0–81.3
C	72.2	68.9–75.3	65.8	61.4–69.9	71.3	68.2–74.1
D	68.7	63.1–73.7	57.6	50.5–64.5	65.7	60.1–70.8
E	74.4	63.4–82.9	80.2	67.4–88.8	72.8	61.3–81.9
Number of NCD	0.455^b^		0.002^b^		0.021^b^	
1	73.1	68.9–76.9	72.8	68.5–76.7	74.5	71.1–77.6
2	72.6	69.4–75.6	65.3	60.0–70.2	73.0	69.7–76.0
≥ 3	70.7	67.4–73.8	63.1	57.7–68.2	68.9	64.8–72.8
Number of medicines needed^e^	0.001^b^		< 0.001^b^		0.001^b^	
1	77.3	72.5–81.4	72.0	65.9–77.4	75.8	71.7–79.4
2	73.2	69.1–77.0	74.5	68.9–79.3	76.2	72.6–79.6
3-4	73.0	69.3–76.3	66.9	61.7–71.7	71.7	68.4–74.7
≥ 5	67.6	63.9–71.1	59.9	53.8–65.7	68.1	63.7–72.2
Self-assessment of health	0.001^b^		0.001^b^		0.001^b^	
Very good/Good	74.6	70.6–78.2	73.3	69.7–76.5	76.4	73.6–79.0
Regular	71.9	68.9–74.6	62.7	57.6–67.5	69.7	66.3–73.0
Bad/Very bad	62.6	57.4–67.6	58.0	49.6–65.9	64.4	59.0–69.4

All	72.3	69.5–74.9	67.3	63.7–70.7	72.4	69.7–74.9

SUS: Brazilian Unified Health System; NCD: non-communicable diseases

^a^ Percentage adjusted by sample weights and by post-stratification according to age and sex.

^b^ Pearson’s Chi-squared test.

^c^ In completed grades in school.

^d^ According to the 2013 Brazil Economic Classification Criterion (CCEB 2013) of the Brazilian Association of Research Companies (ABEP). Available from: http://www.abep.org

^e^ Medicines precribed by the physician.

Total availability of medicines was higher for Fprivadas (88.5%) compared to FPFP (67.4%) and to FSUS (45.2%). For FSUS, availability was higher for men; for those who resided in the South and Southeast compared to the Midwest and Northeast; for those who reported having one NCD; for those who needed one medicine; and for those who showed better self-perception of health. For FPFP, reported total availability was higher in the South compared to the Northeast; for individuals who reported having one NCD compared to those who reported having three or more; for those who needed one medicine compared to those who needed three or more; and for those who self-assessed their health as very good or good. For Fprivadas, reported total availability was higher for users of the Southeast compared to the Northeast, with no significant differences for the other variables analyzed (Table 3).

**Table 3:** Availability of all medicines needed to treat non-communicable diseases in adults and seniors in the SUS pharmacies, Popular Pharmacy Program, and private pharmacies, according to socioeconomic, demographic, and health-related variables. PNAUM, Brazil, 2014.

Variable	Percentage of availability of medicines needed^e^					

	SUS pharmacy		Popular pharmacy		Private pharmacy	
		
	%^a^	95%CI	%^a^	95%CI	%^a^	95%CI
Sex	< 0.001^b^		0.294^b^		0.810^b^	
Male	50.0	46.0–54.0	69.2	64.7–73.4	88.7	86.3–90.8
Female	42.7	39.6–45.9	66.4	62.9–69.7	88.4	87.0–89.7
Age group (years)	0.456^b^		0.017^b^		0.166^b^	
20-39	46.5	40.0–53.0	59.1	49.0–68.5	85.9	80.9–89.7
40-59	43.8	40.3–47.3	71.6	67.1–75.6	89.5	87.8–90.9
≥ 60	46.2	42.6–49.9	65.5	62.1–68.8	88.6	87.2–89.9
Educational level^c^	0.923^b^		0.226^b^		0.133^b^	
0-4	45.8	41.4–50.2	64.7	60.4–68.7	87.9	86.1–89.5
5-8	45.5	40.9–50.0	70.8	65.3–75.8	90.3	88.0–92.1
9-11	44.4	40.6–48.2	69.1	64.6–73.3	89.3	87.2–91.1
≥ 12	46.1	40.1–52.2	67.4	59.5–74.5	85.8	81.0–89.6
Region	< 0.001^b^		< 0.001^b^		0.000^b^	
North	41.7	36.0–47.5	71.7	63.6–78.6	88.6	85.8–91.0
Northeast	31.6	27.9–35.6	54.3	48.4–60.0	82.7	79.4–85.6
Southeast	51.8	47.1–56.5	68.1	63.4–72.5	91.1	89.4–92.5
South	45.8	41.9–49.8	74.5	70.0–78.5	89.6	87.4–91.4
Midwest	33.0	28.5–37.9	72.1	66.6–77.0	87.7	85.3–89.9
CCEB^d^	0.499^b^		0.594^b^		0.719^b^	
A/B	44.2	39.9–48.7	68.8	63.4–73.7	88.6	86.3–90.6
C	45.7	42.4–49.0	67.5	64.1–70.7	88.8	87.5–90.1
D	45.7	40.6–50.8	65.9	59.0–72.2	88.0	84.9–90.6
E	38.5	29.9–47.9	58.6	40.7–74.4	85.0	72.0–92.6
Number of NCD	< 0.001^b^		< 0.001^b^		0.042^b^	
1	56.6	52.4–60.6	74.1	70.2–77.7	90.0	88.4–91.4
2	41.7	37.9–45.6	67.5	62.5–72.1	87.9	85.8–89.8
≥ 3	32.6	29.4–35.9	60.5	55.9–64.8	87.2	85.0–89.1
Number of medicines needed^e^	< 0.001^b^		< 0.001^b^		0.633^b^	
1	57.2	52.4–61.8	75.6	70.5–80.0	89.4	87.4–91.1
2	47.4	43.4–51.4	69.1	63.1–74.5	88.7	86.6–90.6
3-4	41.2	37.7–44.8	65.0	60.0–69.7	87.8	85.2–90.0
≥ 5	29.4	25.3–33.8	57.6	52.5–62.6	87.8	85.1–90.0
Self-assessment of health	< 0.001^b^		< 0.001^b^		0.322^b^	
Very good/Good	54.0	50.3–57.6	72.9	69.1–76.5	89.3	87.5–90.8
Regular	39.0	35.9–42.1	63.5	59.6–67.3	88.1	86.3–89.6
Bad/Very bad	30.3	26.1–34.8	56.8	48.6–64.7	86.9	83.3–89.9

Full	45.2	42.2–48.2	67.4	64.4–70.2	88.5	87.3–89.6

SUS: Brazilian Unified Health System; NCD: non-communicable diseases

^a^ Percentage adjusted by sample weights and by post-stratification according to age and sex.

^b^ Pearson’s Chi-squared test.

^c^ In completed grades in school.

^d^ According to the 2013 Brazil Economic Classification Criterion (CCEB 2013) of the Brazilian Association of Research Companies (ABEP). Available from: http://www.abep.org

^e^ Medicines prescribed by the physician.

Acceptability related to pharmacy services was higher for Fprivadas (98.7%) in relation to FSUS (92.2%). For FSUS, we observed higher acceptability for users aged 60 years or older compared to those aged 40–59 years; for residents of the South, but with no significant difference in relation to the Southeast region; and for those who showed better self-perception of health. Users of the FPFP reported lower acceptability in the North compared to the other regions of the country. For Fprivadas, no significant differences were found for the variables analyzed (Table 4).

**Table 4:** Acceptability related to the SUS pharmacy services, Popular Pharmacy Program, and private pharmacy, according to socioeconomic, demographic, and health-related variables. PNAUM, Brazil, 2014.

Variable	Acceptability to the pharmacy services					

	SUS pharmacy		Popular pharmacy		Private pharmacy	
		
	%^a^	95%CI	%^a^	95%CI	%^a^	95%CI
Sex	0.589^b^		0.601^b^		0.500^b^	
Male	92.6	90.5–94.2	98.1	96.7–98.9	98.5	97.7–99.1
Female	92.1	90.5–93.3	97.7	96.4–98.5	98.8	98.3–99.2
Age group (years)	0.008^b^		0.390^b^		0.386^b^	
20-39	91.1	87.1–94.0	95.0	88.4–98.0	98.3	96.6–99.1
40-59	90.8	88.9–92.4	98.3	97.2–99.0	98.6	97.8–99.1
≥ 60	94.0	92.6–95.2	98.1	97.1–98.7	99.0	98.5–99.4
Educational level^c^	0.678^b^		0.077^b^		0.660^b^	
0-4	92.7	91.0–94.0	98.3	97.2–99.0	98.5	97.7–99.0
5-8	92.5	90.3–94.0	98.2	97.1–98.9	99.2	98.6–99.6
9-11	92.1	89.6–94.0	96.5	93.7–98.1	98.6	97.6–99.2
≥ 12	90.7	86.7–93.6	98.8	96.4–99.5	98.8	97.7–99.4
Region	0.001^b^		0.001^b^		0.413^b^	
North	84.2	80.0–87.7	88.9	84.2–92.4	97.1	95.4–98.2
Northeast	88.8	86.4–87.7	96.3	94.3–97.7	98.3	97.4–98.9
Southeast	93.4	91.0–95.2	98.5	96.6–99.4	98.9	98.3–99.8
South	95.1	92.9–96.7	99.0	96.4–99.5	99.4	98.3–99.8
Midwest	90.0	86.4–92.7	98.6	96.4–99.5	98.5	97.0–99.3
CCEB^d^	0.814^b^		0.781^b^		0.466^b^	
A/B	92.9	89.3–95.4	98.1	96.4–99.0	98.9	97.9–99.4
C	92.3	90.6–93.7	97.7	96.3–98.6	98.8	98.3–99.2
D	91.3	88.8–93.3	97.5	95.6–98.6	98.3	96.8–99.2
E	92.1	83.9–96.3	98.6	95.6–99.6	97.1	90.5–99.2
Number of NCD	0.104^b^		0.237^b^		0.075^b^	
1	93.3	91.5–94.7	97.3	94.9–98.3	98.6	97.8–99.1
2	91.0	90.5–94.7	98.4	97.3–99.1	99.1	98.4–99.5
≥ 3	91.8	89.7–93.6	98.2	96.3–99.2	98.6	97.8–99.1
Number of medicines needed^e^	0.948^b^		0.531^b^		0.075^b^	
1	92.3	89.5–94.4	97.3	95.3–98.4	98.5	97.4–99.1
2	92.3	89.9–94.2	97.5	93.6–99.0	98.0	96.3–98.9
3-4	92.6	90.6–94.3	97.8	96.1–98.8	98.8	98.1–99.2
≥ 5	91.9	89.9–93.6	98.6	97.6–99.2	99.3	98.7–99.6
Self-assessment of health	0.001^b^		0.168^b^		0.142^b^	
Very good/Good	98.4	97.5–99.0	98.4	97.5–99.0	99.0	98.2–99.4
Regular	90.3	88.0–92.2	97.3	95.6–98.3	98.3	97.7–98.8
Bad/Very bad	92.2	90.8–93.4	97.3	94.1–98.8	99.2	98.2–99.6

All	92.2	90.8–93.4	97.8	96.9–98.5	98.7	98.3–99.0

SUS: Brazilian Unified Health System; NCD: non-communicable diseases

^a^ Percentage adjusted by sample weights and by post-stratification according to age and sex.

^b^ Pearson’s Chi-squared test.

^c^ In completed grades in school.

^d^ According to the 2013 Brazil Economic Classification Criterion (CCEB 2013) of the Brazilian Association of Research Companies (ABEP). Available from: http://www.abep.org

^e^ Medicines prescribed by the physician.

For affordability, 2.6% of adults and seniors reported having been out of some of the medicines needed in the 30 days prior to the interview due to financial difficulty.

## DISCUSSION

This study shows high prevalence of full access to medicines for NCD in adults and seniors in Brazil (94.3%). The sum of the prevalences of full and partial access (99.5%) indicates that almost all respondents who reported having medical diagnosis and indication for treatment of NCD managed to obtain some of the medicines they needed.

Despite the high prevalence of full access, we observed significant differences between regions, socioeconomic levels, and health conditions. This indicates strengthening of pharmaceutical services in Brazil, but also the need to improve the pharmaceutical services – especially in the Northeast – and the health care provided to the poorest and sickest.

Study on disparity of access^13^ found prevalence of 87.0% for women of higher socioeconomic level and with one or two NCD and lower prevalence for women of lower socioeconomic level and with three or more NCD. Study on access to medicines for NCD with adults and seniors in two regions of the country in 2005^20^ observed global prevalence of access of 84.0%, which, similarly to this study, was lower for adults (81.0%) than for seniors (87.0%). The higher prevalence of full access observed in this study may reflect both improved supply of free medicines for NCD in FSUS and FPFP and improved income of the population.

Study^26^ that analyzed the access to medicines for NCD in five countries of different income levels (low, medium-low, and medium-high) – Uganda, Ghana, Kenya, Philippines, and Jordan – found prevalence of access of up to 50.0%, ranging from 16.0% to 50.0%.

The theoretical model adopted in this study – used for the first time in household survey employing self-reported information – enabled identifying some barriers and significant differences in the independent variables analyzed for the four dimensions, enabling detailed description of the dynamics of access (Figure).

Geographical accessibility to the three models of provision was regular (≈ 70.0%) and with no significant differences between them. Geographic accessibility to FSUS presented different regional disparities in relation to those identified in general analysis of access, showing that the geographic distribution of FSUS can be improved – especially in the North and Midwest – to better meet the needs of access to pharmacy services of the sickest. Accessibility to FPFP presented differences concerning region, socioeconomic level, and health conditions, indicating the need to promote better geographic distribution of the program, especially in the North, Northeast, and Midwest. Study on accessibility to FPFP found similar result in relation to geographic coverage^9^. Accessibility to Fprivadas showed no regional differences. However, as in the case of FPFP, it was better for the richest and less sick. Although regular accessibility represents a potential barrier to access, the high prevalence of full access observed in this study indicates that users of the three services were able to overcome it.

Higher total availability of medicines in Fprivadas compared to FPFP and to FSUS may be related to the fact that, differently from the private pharmacies, those linked to the SUS and to the Popular Pharmacy Program provide more restricted sets of medicines. The FSUS provide the medicines considered essential, that is, those that meet the health needs of each location, municipality, or state. The FPFP also provide two sets of medicines: one with about 100 pharmaceutical products in the public network, another one more restricted and for the treatment of a group of NCD in the network of private pharmacies^24^ ^,^ ^c^ ^,^ ^d^.

However, this result may reflect the low availability of essential medicines in the SUS (58.5%) observed in national evaluation of key medicine availability in SUS basic health units^17^. A population-based study on access to medicines in the SUS^6^ found even lower prevalence (45.8%). Another study on free access to medicines for hypertension and diabetes^21^ showed that the Family Health Strategy was more effective to ensure free access in the Northeast (62.4%) than in the South (39.6%) and than in traditional models of provision. The results for availability of this study show the complexity involved in obtaining access to all medicines for more than one NCD. These people, who usually need a higher number of medicines, seem to be using more than one source of supply to obtain all the medicines they need.

Lower total availability of medicines in the three models of pharmacies in the Northeast shows the persistence of regional inequalities. Better total availability for men in FSUS may be related to lower number of medicines used by men compared to women, as observed in studies on use of medicine in Brasil^3^ ^,^ ^25^.

Although general acceptability to the three models of provision has been high (> 90.0%), regional differences found between the FSUS and the FPFP indicate the need to direct efforts to improve the quality of service in these provision sources, especially in the North and Northeast. As the number of pharmacies participating in the Popular Pharmacy Program is much higher than that of the public network, it is necessary to establish measures to ensure the quality of service to their users^9^ ^,^ ^24^.

The price of medicines, which is determined both by market-related factors and by the affordability of families and governments, represents a barrier to access, because it affects the capacity of individuals, families, and public and private providers to pay for them. In this study, although the financial barrier explains much of the partial and null access, its magnitude was low. This result indicates that the national policy of medicines and of pharmaceutical services implemented in the country since 1999 seems to be achieving its objective of ensuring access to free or affordable medicines for most of the population with NCD. Previous studies reported higher percentages of non-purchasing of prescription medicines due to financial difficulty, suggesting that the situation may have improved in recent years^1^ ^,^ ^6^ ^,^ ^8^ ^,^ ^19^.

Regarding the limitations of this study, in addition to those specific to household surveys, discussed in the methodological article of the PNAUM^18^, one of the limitations was the use of self-reported measures and restricted to the cases of individuals with NCD who already had medical diagnosis and indication of treatment. Thus, lack of access to diagnosis and prescription may have been a barrier to access to medicines, which was not captured in this study.

Another limitation was the fact that only those who used the pharmacy services to obtain the medicines reported at the time of the interview were considered in the assessment of availability, accessibility, and acceptability in each of them. The methodological choice of not using recall period may have generated a selection bias due to no inclusion of individuals who, perchance, had used the services of pharmacies prior to the interview and that could have a perception or assessment that was different from that observed. Acceptability was investigated considering the characteristics of pharmacy services, but not of the products.

In conclusion, full access to medicines to treat the NCD analyzed in this study was very high. Geographic accessibility to pharmacies was reasonable and can improve, especially for the FSUS and FPFP in the North, Northeast, and Midwest. Difference was observed for total availability between the three services investigated, indicating the need to promote improvement in the supply system of SUS pharmacies, especially in the North and Northeast. Users’ acceptability was very good or good for the three services; however, regional differences suggest the need to promote better quality for FSUS in the North, Northeast, and Midwest and for FPFP in the North. Affordability was very good. The theoretical model, applied for the first time in a household survey and exclusively using self-reported information, enabled observing the complexity involved in the dynamics of obtaining access, as well as identifying barriers to access in the dimensions analyzed.

## Footnotes

a Portal da Saúde. Programa farmácia popular do Brasil. Brasília (DF): Ministério da Saúde; 2004 [cited 2006 Sept 6]. Available from: http://portalsaude.saude.gov.br/index.php/o-ministerio/principal/secretarias/sctie/farmacia-popular

b The national team of the National Survey on Access, Use, and Promotion of Rational Use of Medicines (PNAUM) can be found at the PNAUM Web site: http://www.ufrgs.br/pnaum/equipe

c Set of medicines available at the Farmácia Popular Public network: http://portalsaude.saude.gov.br/images/pdf/2014/fevereiro/20/elenco-fp1-20-08-12.pdf

d Set of free medicines in the Farmácia Popular private associated pharmacies: http://portalsaude.saude.gov.br/images/pdf/2014/dezembro/11/rol-medicamentos-SNTP-11122014.pdf
